# Correction: A Genome-Wide Association Study of Upper Aerodigestive Tract Cancers Conducted within the INHANCE Consortium

**DOI:** 10.1371/annotation/9952526f-2f1f-47f3-af0f-1a7cf6f0abc1

**Published:** 2011-04-26

**Authors:** James D. McKay, Therese Truong, Valerie Gaborieau, Amelie Chabrier, Shu-Chun Chuang, Graham Byrnes, David Zaridze, Oxana Shangina, Neonila Szeszenia-Dabrowska, Jolanta Lissowska, Peter Rudnai, Eleonora Fabianova, Alexandru Bucur, Vladimir Bencko, Ivana Holcatova, Vladimir Janout, Lenka Foretova, Pagona Lagiou, Dimitrios Trichopoulos, Simone Benhamou, Christine Bouchardy, Wolfgang Ahrens, Franco Merletti, Lorenzo Richiardi, Renato Talamini, Luigi Barzan, Kristina Kjaerheim, Gary J. Macfarlane, Tatiana V. Macfarlane, Lorenzo Simonato, Cristina Canova, Antonio Agudo, Xavier Castellsagué, Ray Lowry, David I. Conway, Patricia A. McKinney, Claire M. Healy, Mary E. Toner, Ariana Znaor, Maria Paula Curado, Sergio Koifman, Ana Menezes, Victor Wünsch-Filho, José Eluf Neto, Leticia Fernández Garrote, Stefania Boccia, Gabriella Cadoni, Dario Arzani, Andrew F. Olshan, Mark C. Weissler, William K. Funkhouser, Jingchun Luo, Jan Lubiński, Joanna Trubicka, Marcin Lener, Dorota Oszutowska, Stephen M. Schwartz, Chu Chen, Sherianne Fish, David R. Doody, Joshua E. Muscat, Philip Lazarus, Carla J. Gallagher, Shen-Chih Chang, Zuo-Feng Zhang, Qingyi Wei, Erich M. Sturgis, Li-E Wang, Silvia Franceschi, Rolando Herrero, Karl T. Kelsey, Michael D. McClean, Carmen J. Marsit, Heather H. Nelson, Marjorie Romkes, Shama Buch, Tomoko Nukui, Shilong Zhong, Martin Lacko, Johannes J. Manni, Wilbert H. M. Peters, Rayjean J. Hung, John McLaughlin, Lars Vatten, Inger Njølstad, Gary E. Goodman, John K. Field, Triantafillos Liloglou, Paolo Vineis, Francoise Clavel-Chapelon, Domenico Palli, Rosario Tumino, Vittorio Krogh, Salvatore Panico, Carlos A. González, J. Ramón Quirós, Carmen Martínez, Carmen Navarro, Eva Ardanaz, Nerea Larrañaga, Kay-Tee Khaw, Timothy Key, H. Bas Bueno-de-Mesquita, Petra H. M. Peeters, Antonia Trichopoulou, Jakob Linseisen, Heiner Boeing, Göran Hallmans, Kim Overvad, Anne Tjønneland, Merethe Kumle, Elio Riboli, Kristjan Välk, Tõnu Voodern, Andres Metspalu, Diana Zelenika, Anne Boland, Marc Delepine, Mario Foglio, Doris Lechner, Hélène Blanché, Ivo G. Gut, Pilar Galan, Simon Heath, Mia Hashibe, Richard B. Hayes, Paolo Boffetta, Mark Lathrop, Paul Brennan

An author's name on this paper is misspelled. Tõnu Voodern should be listed as Tõnu Vooder.

